# The Use of Infrared Auto Refractometer for Meibomian Gland Imaging

**DOI:** 10.7759/cureus.51503

**Published:** 2024-01-02

**Authors:** Mohammed Alsaab, Abdulrhman S Alsadun, Eyad Alrsheed, Khaled Almutairi, Abdullah Alkhudhayr, Meshari A Alharbi, Waleed Alghamdi, Omar Solyman

**Affiliations:** 1 Optometry, Qassim University Medical City, Buraydah, SAU; 2 Ophthalmology, Qassim University, Buraydah, SAU; 3 Optometry, Qassim University, Qassim, SAU; 4 Ophthalmology, Qassim University Medical City, Buraydah, SAU

**Keywords:** imaging, mgd, meibomian gland, autorefractor, meibography

## Abstract

Introduction

This study proposes the utility of an infrared auto refractometer for meibography and compares miebographs obtained by an auto refractometer to meibographs obtained by a designated meibography machine.

Methods

A prospective observational comparative study of meibographs of patients with clinical signs of meibomian gland dysfunction (MGD) using a designated meibography machine and an infrared auto refractometer. Five masked, experienced interpreters graded the images of the two machines. The Kappa test was used to calculate Intra-rater and inter-rater agreements between the meibography machine and automated refractor grading of meibomian gland dysfunction.

Results

High-quality photos of all 30 eyes delineating the meibomian glands (MG) were successfully obtained with both the meibography machine and the autorefractor. Both methods had a good intra-rater agreement (κ= 0.667 to 0.784, average 0.738). Poor to fair interrater agreement was noticed in the grading of autorefractor images (k= -0.030 to 0.343, average 0.092) and poor to moderate agreements between investigators for meibography machine images (K= -0.016 to 0.420, average 0.173).

Conclusion

A commercially available auto refractometer could capture high-quality non-contact IR digital meibographs.

## Introduction

Meibomian glands, or tarsal glands, are tubulo-acinar sebaceous glands with holocrine functions responsible for the secretion of the lipid superficial layer of the tear film known as meibum [1]. Meibum comprises various lipids, including wax esters, cholesteryl esters, free cholesterol, triacylglycerols, free fatty acids, phospholipids, and sphingomyelins [2]. This lipid layer plays a fundamental role in the vitality of the ocular surface by maintaining the vertical stability of the precorneal tear film, lubricating eyelid movements, and minimizing evaporation of the aqueous layer of the tear film [3]. Meibomian gland dysfunction is defined as a chronic, diffuse abnormality of the meibomian glands, commonly characterized by terminal duct obstruction with or without qualitative or quantitative changes in the glandular secretion [4]. MGD has a global prevalence of about 21.2% to 40% [3,5-8]. In Saudi Arabia, the prevalence of MGD is thought to be twice the world average, affecting about 70-80% of the population [9]. Morphological changes of meibomian glands associated with MGD include atrophy or loss, tortuosity, thickening, and shortening of the gland. These changes are thought to precede functional changes and result in alteration of the tear film, symptoms of eye irritation, clinically apparent inflammation, and ocular surface disease [10,11].

MGD can be studied using various methods, including a slit-lamp examination for lid morphology and gland expressibility, tear film lipid layer thickness, tear osmolarity, interferometry and evaporimetry, and meibography [12]. Meibography provides photographic delineation of the MG morphology using infrared photography. The study of the infrared meibographs helps diagnose and grade MGD [13,14]. Meibography is traditionally performed by stationary designated expensive machines, which limits it to tertiary and research centers. Although autorefractors are bulky and stationary machines, they are widely available in ophthalmology clinics and optometry offices. Herein, we study the utility of infrared auto refractometers for infrared meibography and compare infrared meibographs obtained by infrared auto refractometers to meibographs obtained by a designated meibography machine for the same patients.

## Materials and methods

This prospective observational comparative study was approved by the institutional review board of Qassim University, Almulida, Al-Qassim, Saudi Arabia, and was performed in accordance with all local laws and compliance with the principles of the Declaration of Helsinki. Patients with clinical signs of MGD, including eyelid margin irregularity or telangiectasia, pouting, or plugging of meibomian gland orifices, were included. Patients with excessive eyelid scarring, which may affect the resolution of infrared images, including patients with a history of prior eyelid surgery or eyelid laceration repair, severe trachomatous scarring, chemical injury, or burns of the eyelids, were excluded. A written informed consent was obtained from all study participants. Infrared meibography of one upper lid was performed for patients with a clinical diagnosis of MGD using OCULUS Keratograph ® 5M meibography machine (Oculus, Menlo Park, California) and Nidek AR-1 infrared auto refractometer (Nidek, Gamagori, Aichi, Japan).

**Figure 1:**
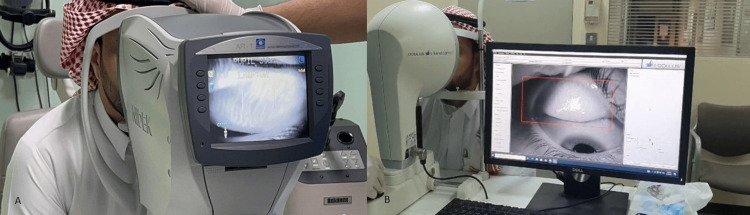
A- a photograph showing the process of capturing meibography images using the A- Nidek autorefractor B- Oculus Meibography machines.

Each participant was properly seated on the autorefractor machine as per regular autorefraction examination, and the upper eyelid of the patient's choice was everted by a cotton-tipped applicator to take the first image (Figure 1A). The chin rest height was adjusted until the upper eyelid of concern was centered in the view. The autorefractometer was manually focused until the morphology of the meibomian glands was in sharp delineation, at which time 3 digital photographs of the autorefractor's screen were captured using an outside smartphone camera (iPhone 13 pro, Apple Inc., Cupertino, California). The patient was then moved and seated on the Oculus Keratograph machine, where a meibograph of the same eyelid was obtained (Figure 1B). The meibographs images obtained from both machines were daintified and coded before they were graded by five masked investigators (an ophthalmologist and four optometrists). A Pult grading system was used for grading the meibographs, in which grade zero was when no change was observed; Grade 1 was considered when there was less than 25% meibomian gland loss. Grades 2, 3, and 4 were considered when there was 26-50% MG loss, 51-75% MG loss, and >75% MG loss, respectively. Interpretation data was fed to the computer and analyzed using IBM SPSS software package version 20.0. (Armonk, NY: IBM Corp). The Kappa test was used to calculate Intra-rater and inter-rater agreements between the meibography machine and automated refractor grading of meibomian gland dysfunction [15]. The significance of the obtained results was judged at the 5% level.

## Results

Thirty upper eyelid meibographs of 30 patients with MGD were successfully obtained using the OCULUS Keratograph ® 5M meibography machine and the Nidek AR-1 infrared auto refractometer.

All 30 autorefractor Miebographs were found to be of high quality and delineated the MG morphology comparable to the designated meibography machine. Masked interpretation of the autorefractor and meibography machine-based images showed a good intra-rater agreement regarding MGD grading with a kappa value ranging between 0.667 to 0.784 with an average of 0.738 (Table 1). A poor to fair agreement was noticed between the investigators in grading of autorefractor-based meibographs (κ = -0.030 to 0.343, average= 0.092), while poor to moderate interrater agreement was noticed for meibography machine-based images (κ = -0.016 to 0.420, average= 0.173) (Table 2).

**Table 1:** Intra-rater agreement between MGD grading for Nidek autorefractor and Oculus Meibography machine-based meibographs Poor agreement < 0.20, Fair agreement 0.21 – 0.40, 0.41 – 0.60 moderate agreement, 0.61 – 0.80 good agreement, 0.81 – 1.00 very good agreement.

Autorefractor vs. Meibography	κ value
Investigator 1	0.784
Investigator 2	0.683
Investigator 3	0.777
Investigator 4	0.667
Investigator 5	0.779

**Table 2:** Inter-rater agreement between MGD grading for Nidek autorefractor and Oculus Meibography machine based meibographs. Poor agreement < 0.20, Fair agreement 0.21 – 0.40, 0.41 – 0.60 moderate agreement, 0.61 – 0.80 good agreement, 0.81 – 1.00 very good agreement.

	Autorefractor	Meibography
	κ value	κ value
Investigator 1 vs. Investigator 2	0.291	0.420
Investigator 1 vs. Investigator 3	0.073	0.164
Investigator 1 vs. Investigator 4	0.066	0.080
Investigator 1 vs. Investigator 5	-0.014	0.039
Investigator 2 vs. Investigator 3	0.099	0.239
Investigator 2 vs. Investigator 4	0.009	-0.016
Investigator 2 vs. Investigator 5	-0.022	0.024
Investigator 3 vs. Investigator 4	0.108	0.270
Investigator 3 vs. Investigator 5	-0.030	0.163
Investigator 4 vs. Investigator 5	0.343	0.345

**Figure 2:**
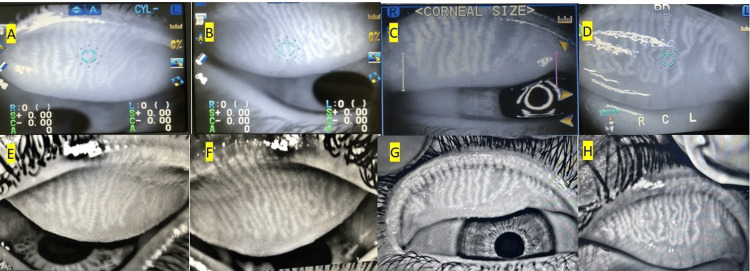
Upper eyelid meibographs using Nidek autorefractor (upper line A, B, C & D) & Oculus Meibography machine (lower line E, D, E & F)

Then, paired images were presented to the investigators without masking, and each of the 5 investigators concluded that the autorefractor-based meibographs were comparable in quality to their meibography machine-based counterparts and reflected the same meibomian gland morphologies in the 30 images (Figure 2).

## Discussion

Meibography is a relatively new imaging technique that describes various methods of visualizing and imaging the meibomian glands. The first description of Meibography by Tapie [16] in 1977 employed endovitreoretinal light probe to visualize the silhouette of the meibomian glands by transillumination through an everted eyelid. The resolution of the meibomian glands was poor. Resolution and delineation of the meibomian glands were improved by the introduction of infrared imaging in 1982 by Jester et al. [17], first described in a rabbit model before it was applied clinically [18]. At that time, the infrared film photography technique was technically challenging and time-consuming. Digital meibography was introduced in 1994 by Mathers et al. [19], who captured digital real-time video meibography videos of transilluminated lids using an infrared camera connected to a computer.

Current non-contact IR meibography was introduced in 2008 by Arita et al. [20]., who describe capturing digital images of the meibomian glands of an everted eyelid using a slit lamp equipped with an IR charge-coupled device video camera which is combined with an IR-transmitting filter. This non-contact method increased patient comfort during the acquisition of the images, making meibography more feasible to perform in clinical practice.

Currently, there are several commercially available meibography machines, including the Keratograph 5M (OCULUS, Wetzlar, Germany), which was used in the current study, and the LipiScan Dynamic Meibomian Imager (TearScience, Johnson and Johnson Vision, Morrisville, North Carolina, USA) [21]. Unfortunately, these devices come at a relatively expensive cost, which limits their availability to large practices and academic centers. In addition, they are bulky and stationary, which limits their use to one place. A cost-effective method of non-contact IR meibography by Pult et al. [22] described using a modified IR security camera for near observation, which was connected to a computer that enabled image capture using generic software.

Another method of addressing the cost limitations of the dedicated infrared meibography machine is by repurposing already available devices. Napoli et al. [23] obtained non-contact IR meibography images using imaging of the everted eyelid by a commercially available spectral-domain optical coherence tomography (OCT) machine (Cirrus™ HD-OCT 4000, Carl Zeiss Meditec Inc., Dublin, California, USA). Because the detection of meibomian glands is difficult or nearly uninterpretable in the raw infrared image on the OCT machine, post-capture modifications of the contrast and brightness were required to increase the visibility of the meibomian glands using the built-in software of the OCT machine [23].

In the current study, we investigated the utility of commercially available autorefractometers for infrared meibography. High-quality infrared miebography images could be obtained from all study participants using the Nidek AR-1 auto refractometer. The quality of the images was comparable in clarity of meibomian gland visualization to the results obtained from the designated meibography machine (OCULUS Keratograph ® 5M). There was a good intra-rater agreement between MGD grading using the meibography machine and the autorefractor (Table 1). However, a poor to-fair agreement was noticed between different investigators. This reflects the subjectivity of the grading system and the big step size (25% step size). However, when investigators were unmasked to the paired images from both machines, all investigators concluded that the images obtained with the two imaging techniques were comparable in resolution and quality.

Autorefractometers are widely available in ophthalmology clinics and optometry offices, which makes meibography readily accessible to a wide range of eye care professionals, compared to OCT machines and commercially designated meibography machines. Moreover, unlike the OCT-dependent techniques, our method did not require any post-processing, thus sparing the operator's time.

Limitations of this technique include the inability to save the images directly on the autorefractor machine or export them to the medical record system. This requires one extra step of external photography of the monitor of the autorefractor by an external camera, like a smartphone camera, for which an assistant may be required. Limitations of this study include the small number of participants. This study aimed to prove the concept of this new technique. We believe that these limitations can be addressed in future studies.

## Conclusions

Repurposing Auto-refractometers to perform meibography is a viable, cost-effective method for morphologically evaluating the meibomian glands. It provided excellent visualization and delineation of the meibomian glands and reflected all the details comparable to the designated meibography machine. Adopting this technique may make meibography accessible to a wide range of eye care professionals worldwide.

## References

[1] Nichols KK, Foulks GN, Bron AJ (2011). The international workshop on meibomian gland dysfunction: executive summary. Invest Ophthalmol Vis Sci.

[2] Butovich IA (2017). Meibomian glands, meibum, and meibogenesis. Exp Eye Res.

[3] Horwath-Winter J, Berghold A, Schmut O (2003). Evaluation of the clinical course of dry eye syndrome. Arch Ophthalmol.

[4] Nelson JD, Shimazaki J, Benitez-del-Castillo JM, Craig JP, McCulley JP, Den S, Foulks GN (2011). The international workshop on meibomian gland dysfunction: report of the definition and classification subcommittee. Invest Ophthalmol Vis Sci.

[5] Shimazaki J, Sakata M, Tsubota K (1995). Ocular surface changes and discomfort in patients with meibomian gland dysfunction. Arch Ophthalmol.

[6] McCann P, Abraham AG, Mukhopadhyay A (2022). Prevalence and incidence of dry eye and meibomian gland dysfunction in the United States: a systematic review and meta-analysis. JAMA Ophthalmol.

[7] Hassanzadeh S, Varmaghani M, Zarei-Ghanavati S, Heravian Shandiz J, Azimi Khorasani A (2021). Global prevalence of meibomian gland dysfunction: a systematic review and meta-analysis. Ocul Immunol Inflamm.

[8] Hom MM, Martinson JR, Knapp LL, Paugh JR (1990). Prevalence of meibomian gland dysfunction. Optom Vis Sci.

[9] Alhamazani MA, Alnabri MS, Alreshidi MN, Alsulaiman HM, Strianese D, Althaqib RN (2021). Assessing public awareness of daily eyelid hygiene habits in Saudi Arabia: an online survey study. Saudi J Ophthalmol.

[10] Robin M, Liang H, Rabut G, Augstburger E, Baudouin C, Labbé A (2019). The role of meibography in the diagnosis of meibomian gland dysfunction in ocular surface diseases. Transl Vis Sci Technol.

[11] Adil MY, Xiao J, Olafsson J (2019). Meibomian gland morphology is a sensitive early indicator of meibomian gland dysfunction. Am J Ophthalmol.

[12] Geerling G, Baudouin C, Aragona P (2017). Emerging strategies for the diagnosis and treatment of meibomian gland dysfunction: proceedings of the OCEAN group meeting. Ocul Surf.

[13] Arita R, Itoh K, Maeda S (2009). Proposed diagnostic criteria for obstructive meibomian gland dysfunction. Ophthalmology.

[14] Eom Y, Lee JS, Kang SY, Kim HM, Song JS (2013). Correlation between quantitative measurements of tear film lipid layer thickness and meibomian gland loss in patients with obstructive meibomian gland dysfunction and normal controls. Am J Ophthalmol.

[15] McHugh ML (2012). Interrater reliability: the kappa statistic. Biochem Med (Zagreb).

[16] Tapie R (1977). Etude biomicroscopique des glandes de meibomius [French]. Ann Oculistique.

[17] Jester JV, Rife L, Nii D, Luttrull JK, Wilson L, Smith RE (1982). In vivo biomicroscopy and photography of meibomian glands in a rabbit model of meibomian gland dysfunction. Invest Ophthalmol Vis Sci.

[18] Robin JB, Jester JV, Nobe J, Nicolaides N, Smith RE (1985). In vivo transillumination biomicroscopy and photography of meibomian gland dysfunction. a clinical study. Ophthalmology.

[19] Mathers WD, Daley T, Verdick R (1994). Video imaging of the meibomian gland. Arch Ophthalmol.

[20] Arita R, Itoh K, Inoue K, Amano S (2008). Noncontact infrared meibography to document age-related changes of the meibomian glands in a normal population. Ophthalmology.

[21] Efron N (2019). 7 - Meibomian Gland Dysfunction. Contact Lens Complications (Fourth Edition).

[22] Pult H, Riede-Pult BH (2012). Non-contact meibography: keep it simple but effective. Cont Lens Anterior Eye.

[23] Napoli PE, Coronella F, Satta GM, Iovino C, Sanna R, Fossarello M (2016). A simple novel technique of infrared meibography by means of spectral-domain optical coherence tomography: a cross-sectional clinical study. PLoS One.

